# A fast vanishing point detection method based on row space features suitable for real driving scenarios

**DOI:** 10.1038/s41598-023-30152-7

**Published:** 2023-02-22

**Authors:** Qin Yang, Yahong Ma, Linsen Li, Yujie Gao, Jiaxin Tao, Zhentao Huang, Rui Jiang

**Affiliations:** School of Information Engineering, Xijing University, Xi’an, China

**Keywords:** Engineering, Mathematics and computing, Computer science

## Abstract

The vanishing point (VP) is particularly important road information, which provides an important judgment criterion for the autonomous driving system. Existing vanishing point detection methods lack speed and accuracy when dealing with real road environments. This paper proposes a fast vanishing point detection method based on row space features. By analyzing the row space features, clustering candidates for similar vanishing points in the row space are performed, and then motion vectors are screened for the vanishing points in the candidate lines. The experimental results show that the average error of the normalized Euclidean distance is 0.0023716 in driving scenes under various lighting conditions. The unique candidate row space greatly reduces the amount of calculation, making the real-time FPS up to 86. It can be concluded that the fast vanishing point detection proposed in this paper would be suitable for high-speed driving scenarios.

## Introduction

In a 3D scene, a set of parallel lines is perspective projected in the 2D image plane of the camera and forms a set of lines in the image plane that intersect at a point, which is called the vanishing point (VP)^1^. The greater the number of parallel lines in the 3D space, the greater the number of vanishing points (VPs) in the corresponding image. VPs can provide a large amount of information existing in the image, so it is involved in many application fields, such as camera localization^2^, camera distortion correction^3^, viewpoint location recognition^4^, Lane Departure Warning (LDW)^5^, and Simultaneous Localization and Mapping (SLAM)^6^. Vanishing points are also used in autonomous driving, and among the many VPs presented in each frame of a driving video, the VPs that require the most attention are the intersections of lanes or road boundaries. VPs provide important information about ADAS and autonomous vehicle driving scenarios. Therefore, the detection of VPs has always been an indispensable factor in autonomous driving. For example, self-driving cars can provide lane departure warnings by identifying drivable areas based on the VPs location. Then, the instantaneous moving direction of the vehicle is judged to assist in determining the position of the lane line. Vanishing point detection techniques for clearly marked structured roads have been widely explored. But vanishing points on unstructured roads are still not marked, which also proves to be a challenging problem^7^.

Existing road VPs detection methods are mainly divided into two categories, traditional edge-based methods, and deep learning-based methods. Traditional edge-based methods in structured and unstructured road VPs detection methods mainly rely on texture features existing in images, with two disadvantages: (1) The detection of edge features is easily disturbed by external conditions, such as uneven illumination, image resolution, and other factors, which can lead to the low accuracy and unstable in many road conditions. (2) The traditional texture detection methods are generally based on a voting mechanism. Due to the increase in the amount of calculation, it cannot meet the real-time conditions required by automatic driving. There have been many strategies using deep learning (DL) to detect VPs. Convolutional Neural Network (CNN) has achieved good results in image classification, image recognition and so on. CNN was used to train the VPs detector in Ref.^8–10^. Regressive ResNet-34 was also used as a vanishing point detector in Ref.^11^. A combination of features from a modified HrNet^12^, plus heatmap regression was applied for VPs detection. Most methods based on deep learning only focus on structured roads, which can be used to detect VPs in restricted driving situations. Furthermore, general supervised learning methods are data-driven learning methods, and large training datasets with precise labels do not exist in real-world autonomous driving scenarios. It can be seen from Ref.^8–10,54,55^ that it is still difficult to achieve VPs detection suitable for real-world complex driving scenarios due to limited training datasets.

The motion-based detection methods^13–16^, this method usually needs to find line segment features from road information, use the intersection of line segments as candidate VPs, and then usually use the threshold method or voting method to filter. There are some shortcomings in these processes, such as the limitation of VPs voting, and a large amount of computation caused by too many motion vectors. In this paper, we propose a fast VPs detection method based on motion-constrained cluster segmentation under row-space features, which can increase detection accuracy and overcome these limitations. Specifically, image features are first extracted and line segments are screened as potential line segments to generate VPs, filter VPs using optimized similarity clustering in line space features, and then use motion vector-based constraints on the extracted points to estimate the most accurate VPs. The innovations of this study are as follows:

1. A processing method under row space features is proposed, which can effectively reduce the global motion vector and reduce the amount of computation.
2. An optimized clustering method is proposed for VPs detection.
3. A motion vector detection linking the upper and lower frames is proposed.
4. The proposed vanishing point detection method clusters candidate VPs in the image by line-space features, and then screens out the wrong VPs by upper and lower frame motion detection. High accuracy and extremely fast speed are achieved in real-world driving scenarios.

## Related work

A lot of excellent work has been done on VPs detection. The current mainstream detection technology is based on image feature extraction, using edges and lines to find candidate VPs in the image, and then using voting methods to determine. Texture-based detection^17–19^, filtering noise by directional filters or steerable filters, followed by edge detection, such as Gabor filters^20^, steerable filter banks^21^. Since the boundaries are somewhat curved, the actual VP cannot be determined, resulting in false detections. Luton et al.^22^ used the Hough transform to detect line segments and determined VP through the intersection of the synthesized line segments. McLean et al.^23^ generated gradient directions by clustering line structures in images, which were validated using two grayscale images. Collins et al.^25^ and Shufelt^24^ used a unique spatial structure to address false edges that have occurred due to orientation constraints. Rother^26^ used the idea of constructing the environment to detect the correct VP in order to avoid errors caused by camera parameters. Cantoni et al.^27^ investigated two methods, by changing the method of line segment detection in images, and by continuous analysis to detect VPs. In particular, deep learning-based methods have recently become very popular for detecting VPs^28,29^, but most current road datasets have little information about vanishing points. This limits deep learning methods that require a large number of training images.

Existing line segment detection methods can be roughly divided into two categories: (1) Hough transform^48–50^; Hough transform is an ingenious feature extraction technique that transforms the global pattern detection problem in the image domain into an efficient peak detection problem in the parameter space. Generally, standard Hough-based line segment detectors first obtain a binary edge map from the input image, use edge operators such as the Canny operator, and then apply Hough transform to the extracted edge map to find all candidate lines, according to a pre-defined gap and length standard, cut it into line segments. (2) Perceptual grouping^51–53^. The perceptual grouping looks at the detection problem from a different perspective. Methods in this class describe line segments as connected regions where sufficiently similar and collinear components exist. The key idea is to generate chains of pixels in a given edge map, extract straight line segments and arcs by traversing these chains and then estimate the area using a validation method. For the first time, a line segment gradient orientation detection is proposed, where a line segment is detected as a rectilinear image region where internal pixels roughly share the same orientation (similarity). This method outperforms the Hough transform in terms of both accuracy and false positives but requires empirical and manual tuning of a range of parameters.

In motion-based vanishing point detection methods^13–16^, corner points are usually taken as the start and end points of motion vectors. At this time, the motion vector can be regarded as a line segment in the video frame. Corner detectors are usually used to detect corners in a video frame, then these corners are used as the starting point, and then the second frame is tracked and used as the endpoint. The start and end points are determined, and the motion vector establishes the connection between the two frames. However, the number of computations increases due to the constant repetition of corner detection and tracking. In addition, the detection of corner points is also unstable. Because the interval between two video frames is short, the motion vector formed by the start and end points will not be very long. The main purpose of image segmentation is to label foreground objects and backgrounds. If objects and backgrounds are properly labeled, key edge information can be obtained by retrieving object contours, which is beneficial for locating vanishing lines and vanishing points. After identifying the vanishing point, the image segmentation also marks the regions of the object and background. This is critical for generating correct depth maps. Clustering algorithms are the most common methods for dealing with image segmentation problems^32–35^. However, it is not easy to correctly label the object and background areas of any image. The image is reproduced by applying the fuzzy c-means algorithm (FCM)^36^ to group the entire pixels according to RGB values. If the object and background are not correctly classified, it means that an accurate initial number of clusters has not been determined, which forms the difficulty of image segmentation. Different initial cluster numbers will produce different segmentation results.

The method used in the current paper is based on line segment detection, it performs vanishing point estimation for clustering under line space features, and then uses a motion-based method to eliminate and limit false vanishing points.

## Vanishing point detection strategy

Vanishing points exist in the intersection of long parallel lines in the image, and lane lines contain many candidate vanishing points. This paper adopts a special spatial feature and improves the clustering strategy, using the similarity theory, the similarity between vanishing points to identify the actual vanishing points, including three steps.

**Figure 1:**
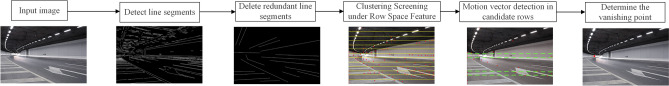
Flowchart of the proposed vanishing point detection.

1. After the line segment is collected, we delete redundant line segments, including short lines, horizontal lines, vertical lines, etc., to reduce the computational cost.
2. Using the spatial features after row segmentation, we perform clustering similarity matching on the candidate vanishing points in the space to optimize the identification of the main vanishing points and perform bubble sorting between row vectors, and the sorting is based on the space of each row. The size of the inner vanishing point cluster similarity; to reduce extreme cases, such as the row that happens to be on the row split, we will extract the top two rows of the ranking as candidate rows.
3. Constrain the motion vectors of the upper and lower frames on the vanishing points in the candidate row and eliminate the noisy candidate points. Figure 1 represents the various stages of the method.

### Line detection and corner collection

**Figure 2:**
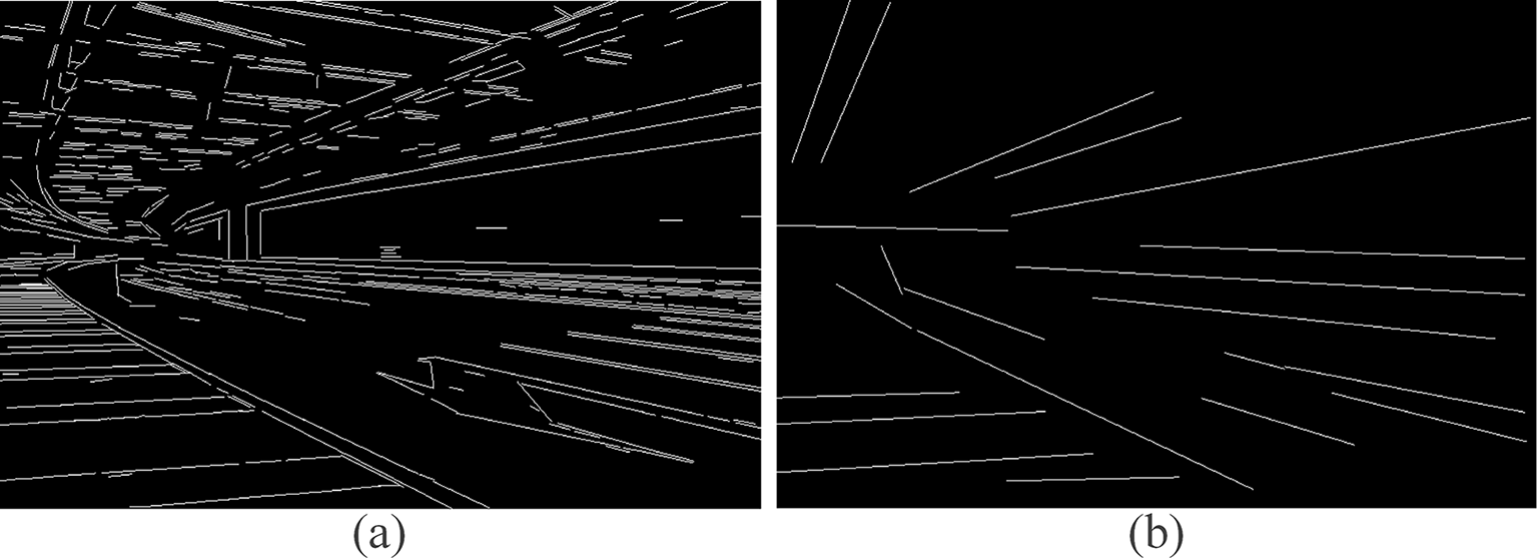
Filter feature line segments.

Image features contain many noisy edges, so detected line segments cannot all be used as feature line segments for estimating vanishing points. It is necessary to filter the parallel line segments on the edge features of the image and delete the redundant line segments. Figure 2 shows the identification of suitable candidate lines. As shown in Fig. 2(a), the Line Segment Detector (LSD) algorithm^37^ is used to collect the line segments contained in the image. In the image, vertical and horizontal lines usually do not belong to the characteristic line segment of the vanishing point, because the point where their line segments meet is often at the edge of the image, so we remove them. As shown in Fig. 2(b), we deleted redundant line segments, and also deleted the shorter lines generated by the distortion, and kept the longer line segments.

**Figure 3:**
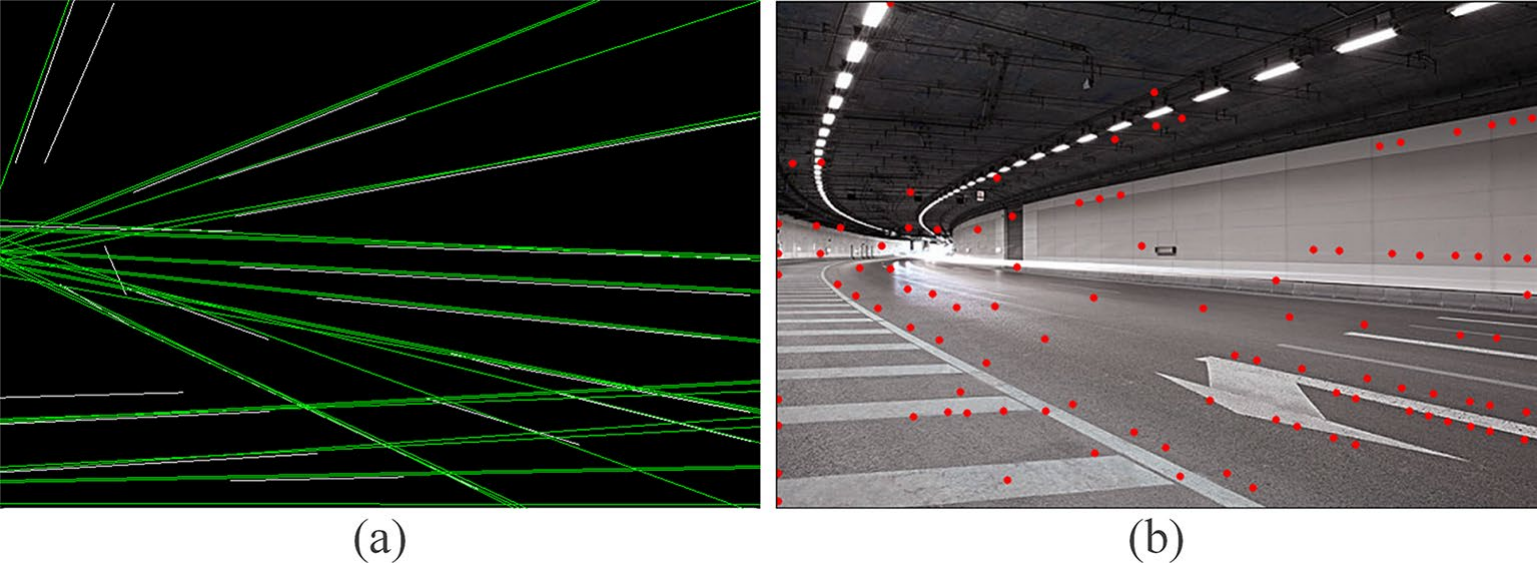
Searching for corners.

Since vanishing points exist at the intersections of feature line segments in the image, the number of feature line segments determines the computation time to find vanishing points. And reducing computation time is also one of the reasons why we filter redundant line segments that are not related to vanishing points. Pass the filtered feature line segments, preserve their directions, and extend their lengths, and utilize the Shi-Tomasi corner detector^30^ to find corners efficiently. All collected corners are taken as an initial set, denoted as *Ft0* = {*Ft0(1), Ft0(2),… Ft0(n),*}, where *n* is the number of corners, as shown in Fig. 3.

### Clustering under row partitioning

The task of dividing data into groups can make objects in a cluster similar in a suitable way.^38^ proposed an idea, transforming the classification knowledge "{Image: Label}" into a pairwise relationship "{(Image A, Image B): Similarity Label}", where similarity represents whether the label of Image A and Image B is the same or not.

Drawing on this idea, the images with the set of corners are clustered, and the task is to filter the vanishing points in each row. This is done by assigning the instance ID of the vanishing point pixel as a unique index to all vanishing point pixels in row space. The indices are integers *i*, 1 ≤ *i* ≤ *n* + 1, where n is the number of vanishing point instances in the scene, add an extra 1 for the best cluster point for the cluster in each row. Create a function *f* that assigns an index y_i_ = f (x_i_) to pixel x_i_, where y_i_ ∈ *Z* and *i* is the index of the vanishing point pixel in the image. The synthetic label of all pixels in the image, i.e., *Y* = { *y*_*i*_ }_∀ *i*_ , should satisfy the relation G. For any two pixels *x*_*i*_*, x*_*j*_*, G(x*_*i*_*, x*_*j*_*) ∈ {0, 1}* is defined as Eq. (1):

1 $$G\left( {{\text{x}}_{i} ,x_{j} } \right) = \left\{ {\begin{array}{*{20}l} {1,} \hfill & {{\text{if}}\;x_{i} ,x_{j} \;{\text{belong}}\;{\text{to}}\;{\text{the}}\;{\text{same}}\;{\text{instance}}} \hfill \\ {0,} \hfill & {{\text{otherwise}}} \hfill \\ \end{array} } \right.$$

*G* is a more accurate representation of the actual clustering target. From Eq. (1), *G* can be directly reconstructed from a given vanishing point. Specifically, if two pixels belong to the same instance, then each output node should not be mapped to a fixed class but should be guided by pairwise similarity. If there are similar pairs, their cluster distribution should be similar and vice versa. The distance between two distributions can be evaluated by pairwise KL-Divergence. Given a pair of pixels *x*_*i*_ and *x*_*j*_, their corresponding output distributions are expressed as X_*i*_ = *f* (x_*i*_) = [t_i,1_..t_i,n_] and X_*j*_ = *f*(x_*j*_) = [t_j,1_..t_j,n_], where *n* is the number of vanishing point indices used for marking. The cost of similar pairs is described as Eq. (2):

2 $$\begin{aligned} &\upzeta \left( {x_{i} ,x_{j} } \right)^{ + } = D_{KL} \left( {X_{i}^{*} \left\| {X_{j} } \right.} \right) + D_{KL} \left( {X_{j}^{*} \left\| {X_{i} } \right.} \right) \\ & D_{KL} \left( {X_{i}^{*} \left\| {X_{j} } \right.} \right) = \sum\limits_{k = 1}^{n} {t_{i,k} log\left( {\frac{{t_{i,k} }}{{t_{j,k} }}} \right)} \\ \end{aligned}$$

The cos*t* ζ (*x*_*i*_, *x*_*j*_)^+^ is symmetric *w.r.t*. *x*_*i*_, *x*_*j*_, where X_i_^*^ and X_j_^*^ are alternately assumed to be constant.

If *x*_*i*_, *x*_*j*_ come from different pairs, the formula shown in Eq. (3) can be used to describe the output distribution of different instances*.*

3 $$\begin{aligned} &\upzeta \left( {x_{i} ,x_{j} } \right)^{ - } = L_{h} \left( {D_{KL} \left( {X_{i}^{*} \left\| {X_{j} } \right.} \right),\sigma } \right) + L_{h} \left( {D_{KL} \left( {X_{j}^{*} \left\| {X_{i} } \right.} \right),\sigma } \right) \\ & L_{h} (e,\sigma ) = max(0,\sigma - e) \\ \end{aligned}$$

Margin σ was set to 2. A criterion is established to evaluate the *G* function and its suitability. By comparing the form, ζ (*x*_*i*_, *x*_*j*_) can be described by Eq. (4), where the integer 1 is used to denote a similarity pair.

4 $$\begin{aligned} & L_{lc} = \zeta (x_{i} ,x_{j} ) \\ & \zeta (x_{i} ,x_{j} ) = G(x_{i} ,x_{j} )\zeta (x_{i} ,x_{j} )^{ + } + \left( {1 - G\left( {x_{i} ,x_{j} } \right)\zeta \left( {x_{i} ,x_{j} } \right)^{ - } } \right) \\ \end{aligned}$$

**Figure 4:**
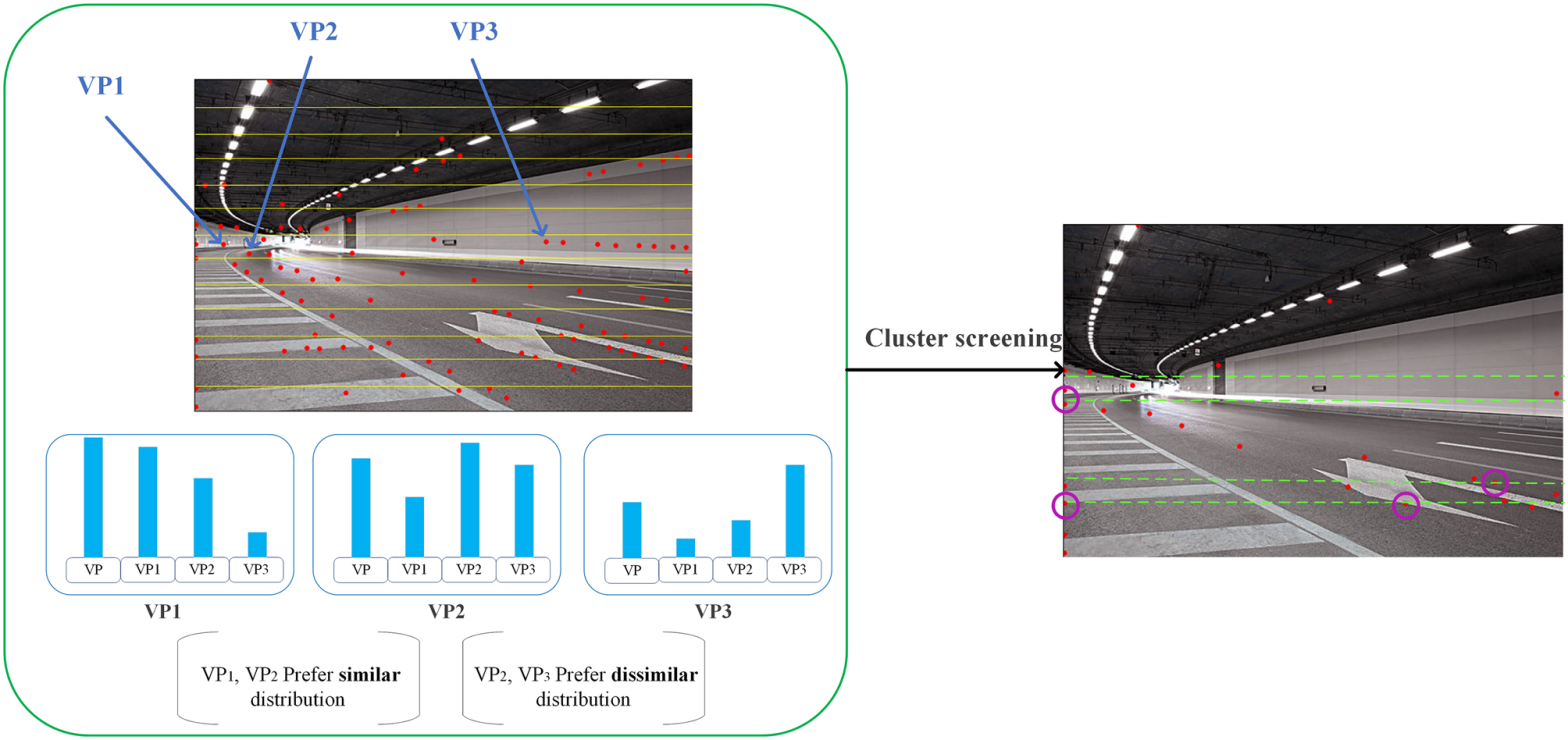
Clustering in row space.

Some problems occur during instance segmentation. Since the vanishing point of the instance is very sparse in the background, and some vanishing points will just be stuck at the edge of the line space feature, where is the vanishing point pixel on the boundary. Although it still belongs to the vanishing point, it is easy to be ignored. According to the resolution of different pictures, it is horizontally divided to form a row space. The first row is selected from the place where the lane lines start in the image as the bottom, and if the image resolution changes, the rows are divided by the principle of equal distribution of resolution. Figure 4 is divided into 13 rows according to the total number of pixels, and each row is clustered. This change not only reduces the difficulty of each row clustering but also improves the computational efficiency. A basic VP cluster point is formed in each row, and the similarity between each instance and the cluster point is analyzed by selecting different vanishing points in the same row. By comparing the output distribution with the cluster points, they can be divided into similar pairs and dissimilar pairs on this basis.

After clustering filtering for each row, the number of candidate vanishing points in each row is compared by bubble sorting. To solve the problem of candidate vanishing points on the boundary, the first two rows will be selected as candidate rows for the next step of motion vector screening. The first two rows here are the first two rows of space after bubble sorting. Since it is impossible to determine whether the VP of the current frame is on the boundary of the row partition, the space of the first two rows of the ranking is chosen to reduce the error arising from this situation. We use purple circles to mark the cases where there are vanishing points on the boundary in Fig. 4.

### Selection of motion vectors

Our proposed detection method takes a given set of clustered corner points *Gt*_(*0*)_ as the starting pixel coordinates of motion vectors bit. Starting from the first frame given at time *t*_*0*_, use the Kanade-Lucas optical flow method^31^ to track each of the initial sets over successive frames *t*_(*0*+*L*)_ (L = 1, 2, 3, …) point. Points not tracked in the initial set in the current frame *t*_(*0*+*L*)_ are removed from the set *Gt*_(*0*+*L-1*)_. Otherwise, update the continuous tracking points into the tracking set *Kt*_(*0*+*L*)_. The corresponding tracking points in the upper and lower frames, *Gt*_(*0*+*L*)_[*i*] and* Kt*_(*0*+*L*+*1*)_[*i*], indicate that their pixel coordinates are *Ut*_(*0*+*L*)_ (*x*_*i*_, *y*_*i*_) and *Vt*_(*0*+*L*+*1*)_ (*x*_*i*_, *y*_*i*_).

**Figure 5:**
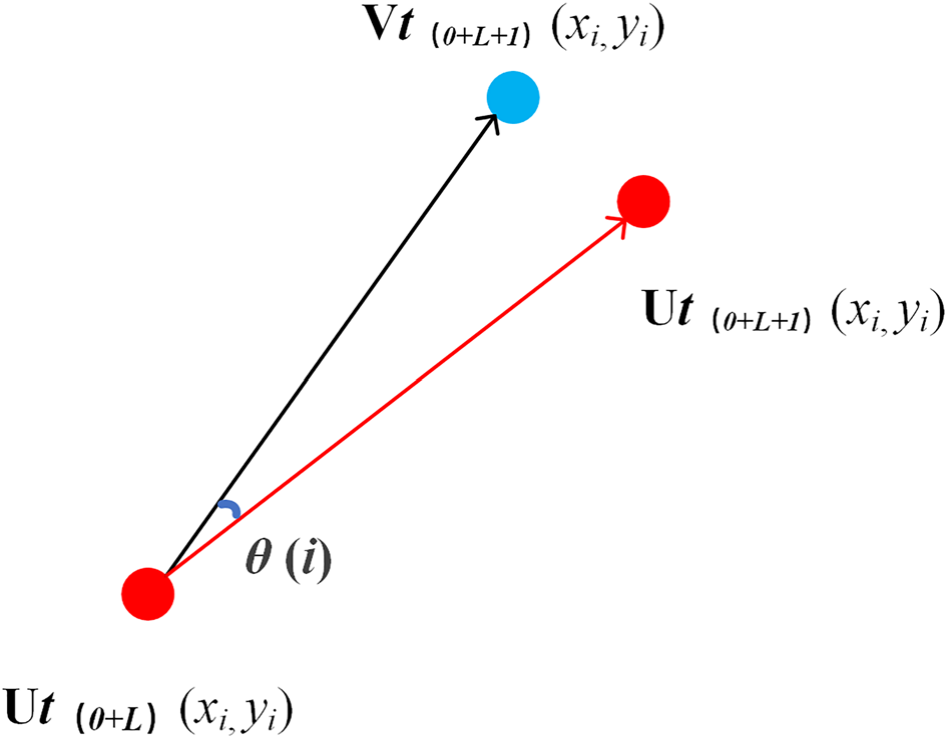
Motion vector filtering vanishing point in line space.

At the same time, the left side of the pixel corresponding to *Gt*_(*0*+*L*+*1*)_[*i*] is represented as *Ut*_(*0*+*L*+*1*)_(*x*_*i*_, *y*_*i*_). At this time, the three coordinates are shown in Fig. 5.

Suppose *Ut*_(*0*+*L*)_(*x*_*i*_, *y*_*i*_) represents the coordinate of the* i*-th candidate vanishing point after the first frame clustering screening, *Vt*_(*0*+*L*+*1*)_(*x*_*i*_, *y*_*i*_) represents the coordinate of the* i*-th candidate vanishing point tracked by the optical flow method in the second frame, and *Ut*_(*0*+*L*+*1*)_(*x*_*i*_, *y*_*i*_) represents the coordinate of the *i*-th candidate vanishing point after the clustering screening of the second frame.

In Fig. 5, the black line represents the motion vector *Z*(*i*) obtained by the optical flow method, and the red line represents the motion vector *C*(*i*) obtained by cluster screening, and the angle between the two lines is *θ*(*i*).

5 $$\theta (i) = \arccos \left( {\frac{Z(i) \cdot C(i)}{{\left\| {Z(i)} \right\|\left\| {C(i)} \right\|}}} \right)$$

when the vector *C*(*i*) is closer to* Z*(*i*), the angle *θ*(*i*) is smaller, and it is used as a metric to establish a piecewise function. When the actual angle *θ*(*i*) is smaller than our set angle threshold, we can set the corner of the upper and lower frames of *C*(*i*) as the vanishing point; otherwise, the index *i* is removed from the set of upper and lower frames.

By this method, the corner points that are misjudged by clustering can be effectively eliminated, and the number of candidate point sets can be continuously limited, and only two candidate rows are used to screen motion vectors, which greatly improves the performance.

## Experiments

In this paper, the widely used datasets are deployed for experiments and analysis, and the experimental results are obtained. Section 1 is an introduction to the experimental setup. Section "Related work" presents the comparison of detection results and the analysis of the proposed method.

### Experimental setup

All experiments were configured on a standard 12th Gen Intel(R) Core (TM) i7-12700H 2.70 GHz computer with 16 GB RAM, implemented via python. Because there are very few structured road datasets with vanishing point labels, some public datasets of unstructured roads are added for experiments on environmental changes such as color and background, AVA Landscape^39^, SIFT flow dataset^40^, all meet the requirements. The AVA dataset, containing approximately 250,000 images, uses 674 images with Vanishing point tags. the SIFT flow dataset consists of 102,206 frames from 731 videos, mostly from street scenes. Ten videos about streets were randomly selected with a total of 1398 frames. 2047 images are transformed for night scenes using CycleGAN. To simulate the detection of actual road information, we join the Jiqing Expressway dataset^41^, which contains various road conditions in the expressway. The dataset contains 32 videos, and each video consists of 5393 frames. This dataset annotates the coordinates of the lane lines, but they are not vanishing point coordinates, so this paper will calculate the intersection of the lanes and use it as the vanishing point ground truth.

We refer to the proposed method as LCS for short. In the comparative experiments of the LCS method, 200 corner points (n_*t0*_ = 200) are detected and tracked in the initial frame. Whenever the number of detected corners is lower than 40, in order to guarantee the number of corners, an additional 100 corners in a new frame are added to the set F_*t*(*0*+*L*)_. In the stable motion vector detection step, when the angle between two consecutive frames is less than a threshold, a tracking point is reserved.

To adjust the parameters required for the experiment, including the Gaussian filter *s* in line segment detection, quantization error specification *q,* and clustering σ, the most suitable parameters are determined by using the clustering score as a consideration index. The line segment sampling rate is related to the detected line segment quality and the number of short line segments (redundant), and the clustering parameter sigma is related to the actual clustering effect. The Normalized Mutual Information (NMI), Adjusted Rand index (ARI), and adjusted Mutual Information (AMI) are used for reference.

The clustering parameter sigma is σ, which can be set to 1 or 2. α = 1 is used to represent the default parameter set for line segment detection, and α = 2 is used to represent the adjusted parameter set with the least redundant line segments, where *s* = 0.1, *q* = 1.4. It can be seen from Table 1 that when using the line segment detection parameter set we adjusted and the clustering parameter σ is 2, the three clustering indicators all reach the best.

**Table 1:** Clustering scores set experimental parameters.

Clustering Scores	NMI	ARI	AMI
α = 1, σ = 1	0.823	0.819	0.864
α = 2, σ = 1	0.876	0.828	0.875
α = 1, σ = 2	0.914	0.852	0.908
α = 2, σ = 2	**0.959**	**0.941**	**0.925**

Significant values are in bold.

### Performance evaluation

The LCS method proposed in this paper is compared with 6 existing vanishing point detection methods of different types. They are the edge-based method^42^, motion-based Road vanishing point detection R-VP^43^, as well as the classic methods of Kong (Gabor)^44^ and yang^45^, the deep learning-based CNN method HrNet^12^, and the disappearance of MST clustering point detection method Hwang^47^.

To evaluate the accuracy of the vanishing point detection method, the normalized Euclidean distance proposed in^46^ is adopted, where the Euclidean distance between the detected vanishing point and the ground truth determined by the diagonal of the input image line lengths is normalized as follows:

6 $$D_{norm} = \frac{{\left\| {U_{d} (x_{i} ,y_{i} ) - U_{g} (x,y)} \right\|}}{DiagLen}$$

where *U*_*d*_(*x*_*i*_,* y*_*i*_) and *U*_*g*_(*x*, *y*) are the coordinates of the detected vanishing point and ground truth, respectively. *DiagLen* is the diagonal length of each image. The value of *D*_*norm*_ depends on whether the position of the vanishing point is close to the position of the ground truth, when it is 0, it proves that the vanishing point coincides with the ground truth.

**Figure 6:**
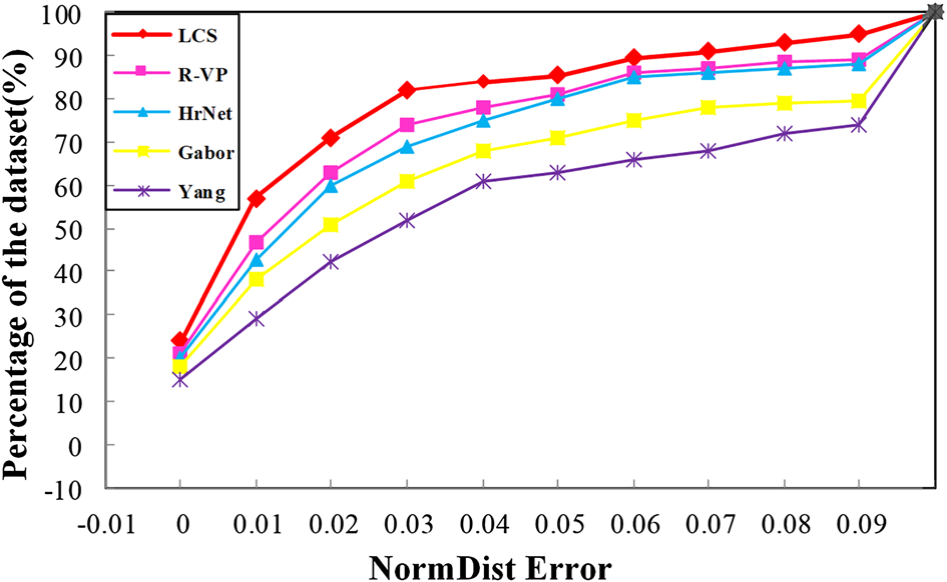
The performance VS normal distance error of different methods.

For each image, calculate the normalized Euclidean distance between the detected ground truth and the vanishing point location. When the detection distance does not exceed the threshold, the detected vanishing point is considered as a correct detection. The performance of different methods can be obtained by changing the threshold from 0 to 0.1 as used in^45^, as shown in Fig. 6.

In Fig. 6, the abscissa represents the normalized distance from the ground truth, and the ordinate represents the percentage of correct predictions. The curves show that our proposed vanishing point detection method is better. Compared with the four vanishing point detection algorithms in the figure, our method exhibits the highest predicted probability in [0, 0.1), and when the normalized Euclidean distance threshold reference is 0.05, the detection accuracy is 4.4% better than the motion-based R-VP method, 5.4% better than the convolution-based HrNet method, 16.3% better than Kong (Gabor) method, and 21.4% better than Yang's method.

The accuracy of the proposed method and the other 4 methods are shown in Table 2, which shows the average value of the normalization error and provides the processing time in milliseconds for comparing the computational complexity. To evaluate the effectiveness of normalization error, all experiments are carried out in the same environment.

**Table 2:** performance comparison in terms of average distance and processing time between ground truths for various methods.

Methods	Average of distance error	Average of processing time (ms)
Edge-based method	0.0191347	611.20
R-VP	0.0038549	62.57 (65fps)
Kong (Gabor)	0.040639	20.1021(s)
HrNet	0.034875	202.4 (33fps)
Proposed	**0.0023716**	**34.29 (86fps)**

Significant values are in bold.

As shown in Table 2 the comparison of edge-based methods with motion-based R-VP, Gabor-based Kong, and convolutional neural network-based HrNet with the proposed LCS is carried out. LCS vanishing point detection applies both clustering and motion vectors, with the highest accuracy (minimum mean error). The proposed method also has excellent processing time. Among them, the FPS measurement can reach 86 when the resolution is less than HD (1280 × 720). The proposed method achieves an average distance error of 0.0023716 (5.06 pixels).

As shown in Table 3, the performance of the threshold method and the perpendicular method under three light environments is compared. The data for bright days and tunnels are extracted from the Jiqing Expressway dataset and evaluated by the average error. Since nighttime videos are not included in the real dataset, additional tests are performed on nighttime scenes. As shown in Table 3, under three different lighting conditions, the metric error using the angle threshold is smaller than that of the vertical distance, and the error of the threshold method is very similar. This demonstrates the generalizability of the proposed method.

**Table 3:** Comparison of the proposed method under three lighting conditions.

	Bright days	Dark days	In tunnels
The proposed method uses an angle-based metric	0.0022184	0.0024741	0.0027047
The proposed method uses the perpendicular distance metric	0.0037955	0.0038219	0.0046192

Ablation experiments are performed on the proposed method, and we use three different line segment detection methods, including Hough line segment detection, LSD, and Canny edge detector. At the same time, vanishing point detection based on the MST clustering method Hwang^47^ is added for clustering comparison. In order to compare the clustering effect, a certain proportion of unstructured road AVA landscape dataset and other datasets are added as support. For experimental accuracy, we use mean squared error (MSE) for evaluation. MSE is the mean of the mean distance of the vanishing point from the ground truth.

As shown in Table 4, the vanishing point detection method using similarity clustering based on LSD detection and optimization is better than other methods. The Hough transform has high time and space complexity, and only straight lines can be determined during the detection process; furthermore, the orientation and length information of the line segment is lost. The Canny edge detector extracts gradient features, but sometimes there are high gradient features that affect cluster convergence and find the best vanishing point because they usually contain a lot of noise. Table 4 shows that the method using LSD edge detector and similarity clustering outperforms other combinations of edge detection and clustering methods.

**Table 4:** Effect comparison of different methods.

Method	MSE (pixel)
The proposed method using LSD	**8.3219**
The proposed method using Canny edge	11.5467
The proposed method using Hough	13.6225
Hwang	11.7184

Significant values are in bold.

**Figure 7:**
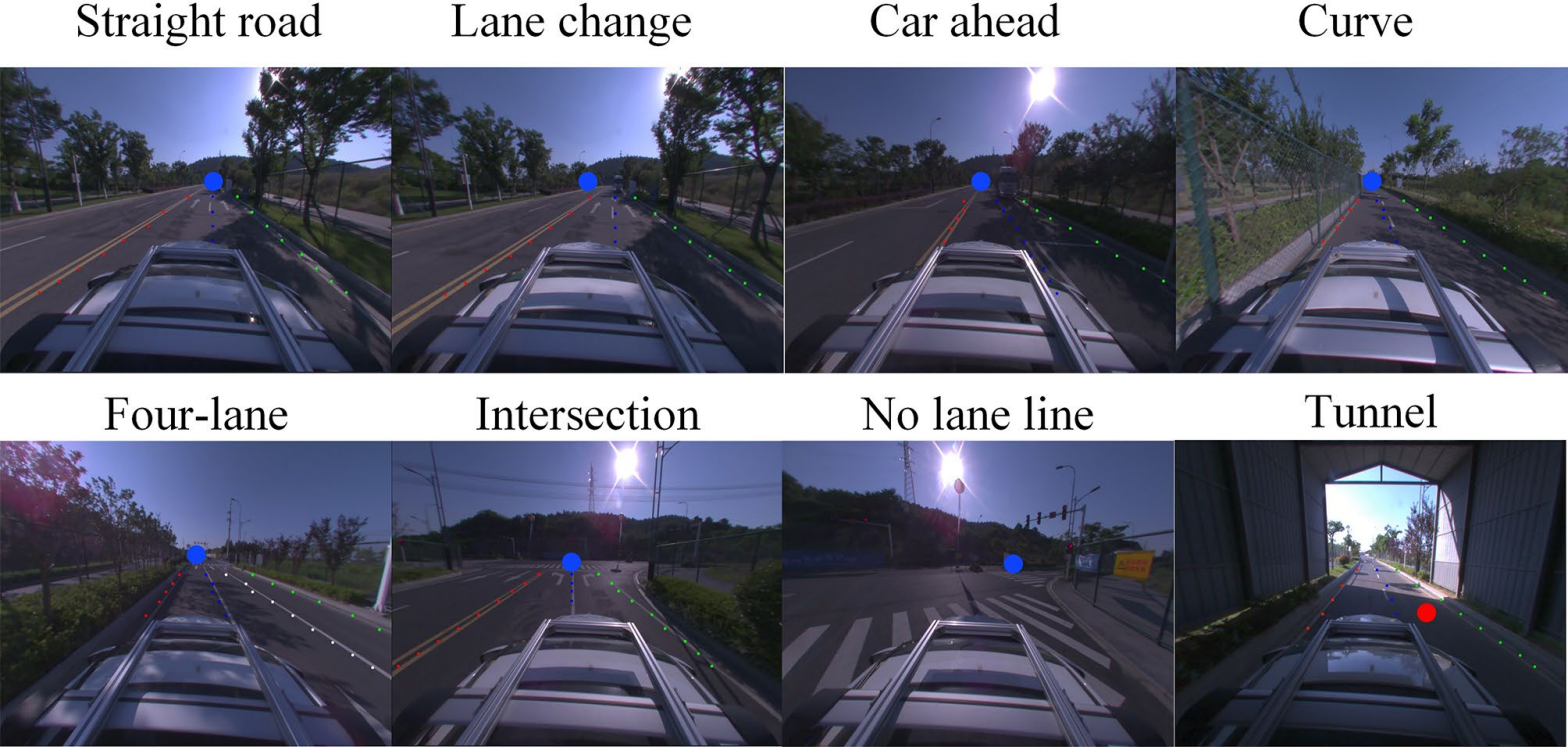
Vanishing point detection under eight road conditions.

To verify that the proposed algorithm can adapt to the requirements of autonomous driving, we use a real car model (BYD Qin) provided by Autocore.ai. The onboard computer was modified to combine AGX Xavier with the car, and the algorithm was transplanted into the real car for real-time testing on the test road in Lishui District, Nanjing City, Jiangsu Province, China. Figure 7 shows the results of driving under different road conditions using our method, which gives an example of vanishing point detection.

Some examples of the proposed method are shown in Fig. 7, and the examples show that the method works well in various road conditions, but the tunnel situation suffers from the phenomenon of vanishing point drift. Note that the vanishing point in the road condition of the tunnel in Fig. 7 is marked in red, indicating that the test failed. The analysis is that due to the large light source difference in the tunnel, the cluster similarity cannot be correctly matched. The remaining seven regular road conditions were observed that the detected vanishing points overlapped visually with the intersection of the road boundary, proving the accuracy of the proposed method. The robustness and generalization of the proposed method can be seen in the actual measurement of seven conventional road conditions.

**Figure 8:**
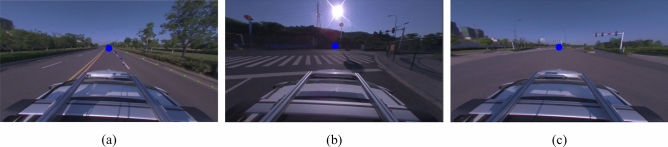
Vanishing point detection in curved situations.

The biggest role of the vanishing point is to delineate the drivable area for the automatic driving system. There are three types of curves, the first one is a regular curve as in Fig. 8(a). The second type is the premise of excluding the traffic rules, when the curve is driven, the area centered on the vehicle is drivable. At this time there is no restriction on the driveable area, so the vanishing point will exist directly in front of the front of car when the curve scenario is as in Fig. 8b. Another situation is when in a large intersection, as in Fig. 8c, the vanishing point will be in the closest road position to the direction of vehicle travel.

In general, from observations on structured road datasets (Nanjing Road Survey and Jiqing Expressway datasets) and unstructured road datasets (AVA landscape and SIFT flow datasets, etc.), the method can effectively detect vehicles When to drive the vanishing point normally; however, when encountering a tunnel, the vanishing point sometimes cannot be detected correctly.

## Conclusion

In this paper, we investigate a VP detection method based on similarity clustering and motion constraints under row space features. Filter out redundant lines to collect corner points, perform similarity clustering in the row space, and only limit the motion vectors of the corner points that meet the conditions in the candidate row, which greatly reduces the amount of calculation. The FPS detection rate can reach 86, which can fully meet the requirements of real-time detection of autonomous driving.

The method has been verified by experiments and can quickly find the vanishing point even when there is no prior information on scene and camera parameters in the environment, and there are problems such as partial occlusion, background clutter, viewpoint changes, and lighting effects. Situations that cannot be detected correctly are only for the tunnel situation that occurs on structured roads. This shows that the proposed method is both robust and accurate. From the computational complexity point of view, the proposed approach can be implemented in real-time, enabling fast detection while ensuring accuracy. Our method plays an important role in drivable area detection, improving the accuracy of lane line detection, and autonomous driving applications.

Future work will explore how to overcome tunnel conditions and identify drivable areas with vanishing points, assisting lane line detection for area detection.

## Data Availability

All data generated or analyzed during this study are included in this published article (and its Supplementary Information files).
